# Impact of childhood traumas and early substance abuse in deviant behaviors

**DOI:** 10.1192/j.eurpsy.2025.1980

**Published:** 2025-08-26

**Authors:** M. Gambini, D. Marazziti, A. Arone, V. Caruso, F. Weiss, A. Coccoglioniti, G. Russomanno, L. Foresi

**Affiliations:** 1 Department of Clinical and Experimental Medicine, University of Pisa, Pisa, Italy

## Abstract

**Introduction:**

Youth violence, particularly among men, is a significant global issue. The primary causes leading to detention orders are drug-related crimes, armed robberies, and terrorism. In addition to social and environmental factors, early attachment experiences and stressors can significantly affect lifelong development, especially during adolescence, impacting learning, behavior, emotions, and health. Thus, identifying underlying traits of chronic deviant behavior, such as attention-deficit/hyperactivity disorder (ADHD) and drug, may help clarify the mechanisms driving different problem behavioral disorders throughout life and be crucial in structuring effective prevention programs for these people.

**Objectives:**

This study aimed to examine clinical and psychopathological features in imprisoned young adults with comorbid substance use disorder (SUD).

**Methods:**

The study was conducted in Colombia at a detention center for young adults who committed serious crimes during adolescence. Forty participants with SUD were included and assessed using six questionnaires and clinical interviews, aiming to examine their clinical and psychopathological characteristics.

**Results:**

The mean age of initial drug contact was 12 years, and the most consumed drugs were cannabis, alcohol and cocaine/amphetamines. Significant positive correlations were found between emotional dysregulation and hyperactivity (p=0.010), inattention (p=0.003), executive dysfunction (domains of inhibition (p=0.001) and initiative (0.001), mood swings (p=0.027) and childhood trauma. Individuals with psychostimulant substances disorder showed a significant positive correlation with the “Authority” domain (p=0.043), and those using sedative substances also with the “Resentment/Self-Sufficiency” and “Violent Childhood” domains.

**Conclusions:**

Our findings suggest that greater impulsivity during childhood exposure to violence represents a predictor of emotional dysregulation, with a large impact later. Impulsivity and difficulty accepting negative emotions are positively associated with executive dysfunctions, especially in inhibitory control, emotional regulation, and initiative. A permissive childhood, characterized by a lack of guidance and boundaries, can be a predictor of committing murder later in life. Taken together, these findings highlight the

**Disclosure of Interest:**

None Declared

